# Estimating non-additive within-season temperature effects on maize yields using Bayesian approaches

**DOI:** 10.1038/s41598-019-55037-6

**Published:** 2019-12-06

**Authors:** Jisang Yu, Gyuhyeong Goh

**Affiliations:** 1 Department of Agricultural Economics, Kansas State University, Manhattan, KS66506 USA; 2 Department of Statistics, Kansas State University, Manhattan, KS66506 USA

**Keywords:** Climate-change impacts, Climate-change impacts

## Abstract

Detrimental impacts of extreme heats on the U.S. crop yields have been well-documented by a number of empirical studies. However, less have focused on within-growing season weather variation and the interaction between temperature and precipitation. The objective of this study is to emphasize the importance of disaggregating temperature exposures within growing season. To achieve our objective, we estimate the impact of within-season monthly temperature and precipitation variations on maize yields in the U.S. corn belt region. We provide a discussion on variable selection methods in the context of estimating crop yield responses to climate variables. We find that the models that utilize within-growing season monthly variations performs better compared to the models with growing season aggregated weather variables and show the strength of Bayesian estimations. We also find that the warming impacts predicted by the models that utilize within-growing season variations are smaller than the predicted impacts of the models with aggregated weather variables. The findings indicate that the temperature effects are not additive across months within growing season.

## Introduction

In the context of investigating the effects of climate change and global warming on food production, a number of empirical studies provide empirical evidence on detrimental impacts of extreme heats on the U.S. crop yields^1–4^. While many of the previous empirical studies implicitly assume the time separability of temperature effects and use annual or growing-season aggregation, several recent studies highlight the importance of within-growing season weather variations^5–8^. Contributing to the recent empirical literature, the objective of our study is to emphasize the importance of utilizing within-growing season weather variations. We show that disaggregating temperature exposures within growing season by month leads to substantially different predictions of crop yields as responses to global warming. To achieve our objective, we estimate the relationship between temperature and precipitation variations and maize yields in the U.S. corn belt region with several different specifications and describe the role of the interaction between temperature and precipitation on maize yield response models. We also compare the out-of-sample prediction performances across different specifications and estimation methods.

As observed by the recent studies^5,6,8^, timings of temperature exposures within the growing season play important roles explaining crop yield variations. For example, Butler and Huybers^5^ find more negative effects of extreme heats on the U.S. maize yields during grain filling stage compared to vegetative or drydown stages. For the case of Kansas wheat, temperature effects on yields have substantial variations within the growing season^6^. Water stress is also, of course, crucial for plant growth. Although several previous studies^1,9^ find empirical evidence on smaller impacts of precipitation compared to temperature explaining the U.S. maize yields, a recent study^10^ find that about 13% of yield variability is due to meteorological drought. Water availability also interacts with temperature and affects the yield sensitivity. Extreme heat has a smaller detrimental yield effect when there is more available water^11^. In the case of Kansas wheat, the estimates of Tack *et al*.^6^ indicate that the detrimental impact of warming would be reduced with greater rainfall in Spring. Also, recent empirical studies^12,13^ find that irrigation would offset the detrimental heat impacts. The recent studies highlight the importance of interaction variables between temperature and precipitation. Therefore, we utilize monthly degree day and precipitation variables and interaction variables between degree days and precipitation to explain yield variability.

Introducing monthly weather variables and the interaction terms among these variables leads to a substantial number of explanatory variables. It is well-known that the ordinary least squares (OLS) estimates lead to poor estimation and prediction accuracy with a large number of explanatory variables^14^, often referred to as the curse of dimensionality. As an alternative to the OLS estimation, penalized least squares (PLS) methods have been developed and employed in recent high-dimensional data analyses; for the recent discussion on PLS, see Wang^15^. While PLS can reduce the high-dimensionality by inducing exact zero values of coefficient estimates for irrelevant predictors, the determination of tuning parameters, which control the degree of the sparsity, is still a difficult  challenge. Over the last several decades, many studies show that Bayesian approaches can provide effective ways for high-dimensional data analysis^16–18^. In a Bayesian framework, the tuning parameter selection problem can be resolved by integrating out the tuning parameter through Markov Chain Monte Carlo (MCMC) method. Therefore, we utilize two Bayesian methods, Bayesian variable selection (BVS) and Bayesian model averaging (BMA), to estimate our preferred specification with monthly degree day and precipitation variables and the interaction variables and show that the Bayesian methods perform better in terms of out-of-sample predictions than OLS estimations.

## Results

We use county-level annual maize yields data from Illinois, Indiana, and Iowa that span 1981–2017 and monthly weather data for the same period. Details on data and methods are in the methods section. In this section, we first show how each specification and estimation method perform in terms of out-of-sample predictions and we report the predicted impacts of uniform warming scenarios. We also present the marginal effects of temperatures on maize yields from our preferred specification.

### Comparing model performances

Table 1 provides the descriptions of the candidate models with different sets of explanatory variables. We start with a model that uses aggregated temperature variables, Growing Degree Days (GDD) and Heating Degree Days (HDD), precipitation variables, and quadratic time trends M1^1^. Aggregation is over the growing season, which is from April to October. We define the growing season based on the planting dates and the harvesting dates provided by National Agricultural Statistics Service^19^. For the second model, M2, we add the interaction terms between GDD and precipitation, between GDD and precipitation squared, between HDD and precipitation, and between HDD and precipitation squared, to represent possible interplay among the weather variables. Note that the quadratic term for precipitation captures the nonlinearity of precipitation effects. Each model has state-specific quadratic time trends and county-level fixed effects (*v*_*i*_).

**Table 1:** Four alternative specifications.

Models	Model forms
M1	ln *yield*_*it*_ = *β*_1_*GDD*_*it*_ + *β*_2_*HDD*_*it*_ + *β*_3_*Prec*_*it*_ + ${\beta }_{4}Pre{c}_{it}^{2}$ + ${\sum }_{s}\,{\delta }_{1s}{D}_{s}\times Tim{e}_{t}$ + ${\sum }_{s}\,{\delta }_{2s}{D}_{s}\times Tim{e}_{t}^{2}$ + *v*_*i*_ + *ε*_*it*_
M2	ln *yield*_*it*_ = *β*_1_*GDD*_*it*_ + *β*_2_*HDD*_*it*_ + *β*_3_*Prec*_*it*_ + ${\beta }_{4}Pre{c}_{it}^{2}$ + *β*_5_*GDD*_*it*_ × *Prec*_*it*_ + *β*_6_*GDD*_*it*_ × $Pre{c}_{it}^{2}$ + *β*_7_*HDD*_*it*_ × *Prec*_*it*_ + *β*_8_*HDDit* × $Pre{c}_{it}^{2}$ + ${\sum }_{s}\,{\delta }_{1s}{D}_{s}\times Tim{e}_{t}$ + ${\sum }_{s}\,{\delta }_{2s}{D}_{s}\times Tim{e}_{t}^{2}$ + *v*_*i*_ + *ε*_*it*_
M3	ln *yield*_*it*_ = ${\sum }_{m=4}^{10}\,{\beta }_{1m}GD{D}_{imt}$ + ${\sum }_{m=4}^{10}\,{\beta }_{2m}HD{D}_{imt}$ + ${\sum }_{m=4}^{10}\,{\beta }_{3m}Pre{c}_{imt}$ + ${\sum }_{m=4}^{10}\,{\beta }_{4m}Pre{c}_{imt}^{2}$ + ${\sum }_{s}\,{\delta }_{1s}{D}_{s}\times Tim{e}_{t}$ + ${\sum }_{s}\,{\delta }_{2s}{D}_{s}\times Tim{e}_{t}^{2}$ + *v*_*i*_ + *ε*_*it*_
M4	ln *yield*_*it*_ = ${\sum }_{m=4}^{10}\,{\beta }_{1m}GD{D}_{imt}$ + ${\sum }_{m=4}^{10}\,{\beta }_{2m}HD{D}_{imt}$ + ${\sum }_{m=4}^{10}\,{\beta }_{3m}Pre{c}_{imt}$ + ${\sum }_{m=4}^{10}\,{\beta }_{4m}Pre{c}_{imt}^{2}$ + ${\sum }_{m=4}^{10}\,{\beta }_{5m}GD{D}_{imt}$ × *Prec*_*imt*_ + ${\sum }_{m=4}^{10}\,{\beta }_{6m}GD{D}_{imt}$ × $Pre{c}_{imt}^{2}$ + ${\sum }_{m=4}^{10}\,{\beta }_{7m}HD{D}_{imt}$ × *Prec*_*imt*_ + ${\sum }_{m=4}^{10}\,{\beta }_{8m}HD{D}_{imt}$ × $Pre{c}_{imt}^{2}$ + ${\sum }_{s}\,{\delta }_{1s}{D}_{s}\times Tim{e}_{t}$ + ${\sum }_{s}\,{\delta }_{2s}{D}_{s}\times Tim{e}_{t}^{2}$ + *v*_*i*_ + *ε*_*it*_

We then extend our models to account for within-growing season variations. Our extension is somewhat similar to that of Tack *et al*.^6^ except we utilize monthly variables instead of three seasons. The third model, M3, is the extension of M1 with monthly variables from April to October. Similarly, the last model, M4, is the extension of M2 and is our most preferred model. M4 has 56 weather-related variables, state-specific quadratic time trends and county-level fixed effects, which leads to the concerns on the reliability of OLS estimates.

Therefore, we utilize two Bayesian approaches: BVS and BMA. Both Bayesian approaches use the posterior model probabilities that quantify the importance of candidate models given the observed data. BVS identifies relevant weather variables in M4 by searching for the model with the highest posterior model probability among all possible reduced models. However, selecting a single model, as BVS does, may fail to account for the model uncertainty whereas averaging cross many candidate models with Bayesian weights, i.e. BMA, performs well even with the presence of the model uncertainty (We also estimate M4 with LASSO, which is one of the common PLS approaches, for the comparison, and report the results in the online Supplementary Information)^16^. See the online Supplementary Information for more technical details on our two Bayesian estimation methods, BVS and BMA.

We first discuss the out-of-sample performances of the six specifications. We perform the out-of-sample predictions for each specification by conducting k-fold cross-validation with $k=10$. Details are in the methods section. Table 2 reports the Root Mean Squared Errors (RMSEs), the Mean Absolute Percentage Errors (MAPEs), Pearson’s correlation coefficient (PCC) and the Skill Scores of the six candidate specifications. The formulas for the RMSEs, MAPEs, PCC, and Skill Scores are in the methods section.

**Table 2:** Out-of-sample prediction performances.

Specifications	RMSE	MAPE	PCC	Skill Score
M1	0.1527	0.0236	0.7695	0.0000
M2	0.1555	0.0239	0.7708	−0.0574
M3	0.1501	0.0234	0.8204	0.0284
M4-OLS	0.1411	0.0220	0.8389	0.1218
M4-BVS	0.1262	0.0196	0.8656	0.2832
M4-BMA	0.1265	0.0196	0.8648	0.2772

Note: Skill Scores are computed based on M1 as a benchmark.

As expected, we observe that our preferred model (M4) outperforms other three models regardless of the estimation methods. Based on the Skill Score measures, M4 specifications are performing about 12–28% better than M1. Among three estimation methods, the Skill Scores of Bayesian methods are more than double of that of OLS. Variable selection procedures via Bayesian approaches lead to better out-of-sample prediction performances by avoiding over-fitting. We do not find much difference between BVS and BMA, which suggests that, for the M4 specification and our dataset, BVS does not suffer much from the model uncertainty. Overall, we conclude that disaggregating weather data into finer time scales (i.e. monthly) adds information in terms of explaining the variations in maize yields.

### Impacts of uniform warming

**Figure 1:**
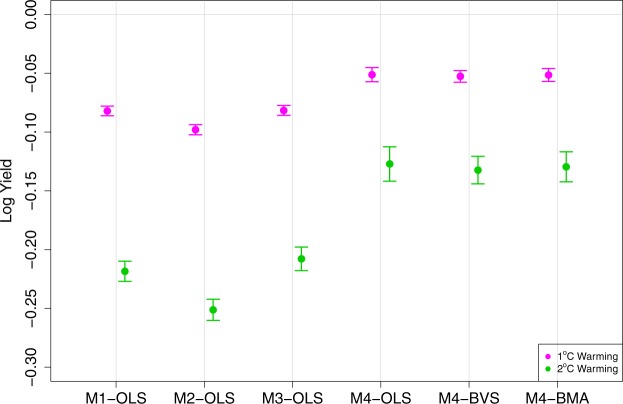
Average impacts of uniform 1 °C warming on US maize yields by specification. Pink represents the average impacts of 1 °C warming and green represents the average impacts of 2 °C warming. Changes are represented in log-scale.

To provide some insights on the importance of model selections in the context of predicting the warming effect on crop yields, we estimated the impact of uniform 1 °C and 2 °C warmings on maize yields for all six specifications. Figure 1 reports the average impact of uniform 1 °C and 2 °C warming represented as changes in logs by specification.

A notable observation from Fig. 1 is that the negative warming effects are substantially smaller when the monthly weather variables are used (M3 and M4) compared to the models with growing season aggregated variables (M1 and M2). This is also lower than the previous estimates^20^. This indicates that the time separability assumption of heat effects may not be appropriate and the aggregation of temperature variables across months leads to the estimates with more negative warming effects. Introducing interaction terms only makes significant difference when monthly variables are used and slightly reduces the detrimental warming effect. The difference between M4 specifications and the other specifications is greater for the 2 °C warming scenario.

Note that the specifications that use the growing-season aggregated weather variables implicitly impose a restriction that the marginal effects of these variables are same across the months. For example, those specifications do not distinguish a unit increase in HDD in April from a unit increase in HDD in July. While the aggregation may lead to the estimates that are approximately average of the monthly effects, the estimated impacts of uniform warming are likely to be significantly different if we have heterogenous marginal effects across months since uniform 1 °C warming would increase HDD for each month in a nonlinear way^21^. Similarly, the interaction effects between temperature and precipitation can be very different across months and the aggregation would only capture the average of them.

We also observe the greater precisions of the estimated impacts from the Bayesian approaches. Compared to M4 - OLS, the confidence intervals are narrower for the estimates from M4 - BVS and M4 - BMA. In other words, the specifications M4 - BVS and M4 - BMA are providing more precise estimates of the impacts of uniform warming on maize yields. Combined with the better out-of-sample prediction performances we observe in Table 2, we believe that the two Bayesian specifications provide reliable estimates of the warming impacts.

**Figure 2:**
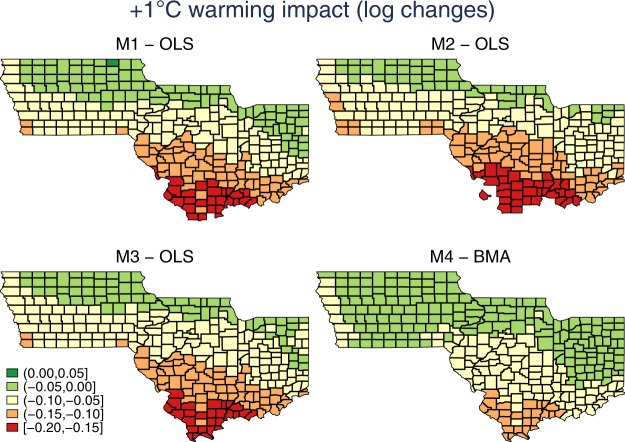
Predicted impacts of uniform 1 °C warming by model (Changes in log-scale).

Finally, we check whether different specifications lead to warming impact estimates with different spatial distributions. Figure 2 presents the county-level predicted yield changes as response to the uniform 1 °C warming by model. As expected, northern regions are less negatively affected by the uniform 1 °C warming. Consistent with Fig. 1, M3 and M4 show smaller negative warming impacts compared to M1 and M2. The ordinal spatial patterns are similar across different models indicating that the differences across models do not vary much across space.

### Marginal effects of monthly temperature variables

While our main objectives are to show a) that specifications with monthly weather variables perform better in terms of out-of-sample predictions and b) that specifications with monthly weather variables predict different warming impacts on maize yields, we also provide the marginal effects of monthly temperature variables to understand what drives the main results.

Since M4 estimated outperforms the other specifications, we limit our focus to the estimates of M4. We report the marginal effects of monthly GDDs and HDDs, which are measured in log changes in yields as responses to a unit changes in GDD and HDD, that are computed based on the estimates from M4 - BMA. We show that the effects are heterogenous across different months, which is a potential source of the differences in warming impact predictions across different model specifications. With the interaction terms among temperature and precipitation variables, we estimate the conditional marginal effects of GDDs and HDDs at high and low precipitation levels, high measured at 75% percentile, and low measured at 25% percentile.

**Figure 3:**
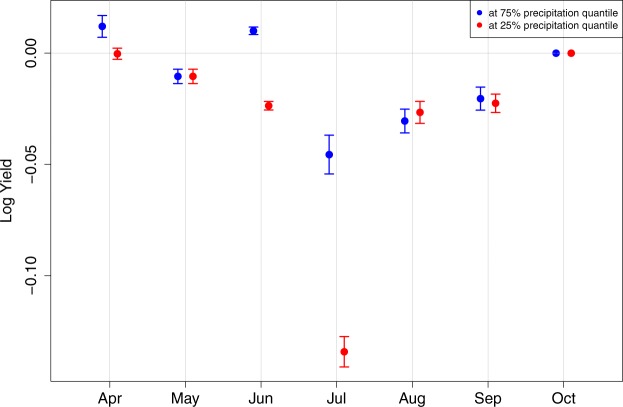
Conditional marginal effects of the HDDs by month estimated by M4 - BMA. Blue represents the conditional marginal effects at the high level of precipitation, 75% percentile, and red represents the conditional marginal effects at the low level of precipitation, 25% percentile.

**Figure 4:**
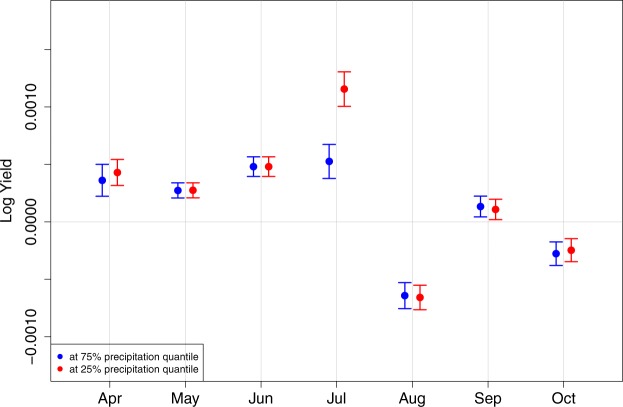
Conditional marginal effects of the GDDs by month estimated by M4 - BMA. Blue represents the conditional marginal effects at the high level of precipitation, 75% percentile, and red represents the conditional marginal effects at the low level of precipitation, 25% percentile.

Figure 3 presents the estimated conditional marginal effects of the HDDs for given precipitation levels. Throughout May to September, HDDs have significant and substantial negative effects on maize yields. The estimates are consistent with agronomy literature that finds the negative impact of heat during late-vegetative stage on maize development through delays in silking^22^ and during grain filling on maize kernel dry weights^23^. We do not observe significant impacts of precipitation on the impacts of HDDs except June and July (see Fig. 3). This can be explained by few previous findings such as smaller detrimental effects of high temperature exposures with greater amount of water availability^11^ and no significant heat impacts on irrigated maize^13^. Although further researches on the simultaneous impact of extreme heat and drought with better measures of water availability are needed (e.g. as in Ortiz-Bobea *et al*.^8^), our findings indicate that extreme heat with low precipitation can lead to greater loss in maize yields during June and July, which cover late-vegetative and early grain-filling stages. Figure 4 reports the estimated conditional marginal effects of the GDDs for given precipitation levels. The estimated marginal effects of GDDs are also heterogenous across months. As expected, we observe that the marginal effects of the GDDs throughout April to July are beneficial to maize yields. While it is outside of the scope of this study to closely examine each marginal effect, we acknowledge that the negative effect of GDD in August are not expected. This may be the result of multicollinearity or imposing same temperature thresholds for GDD and HDD calculation across different months. Future studies are needed to investigate additional models to examine this negative effect.

## Discussion

The main findings of our study are a) the models utilizing disaggregated monthly weather variables outperform the models with aggregated weather variables, b) the Bayesian Modeling Averaging method produces more precise estimates when we have a relatively large number of predictors in the model, and c) the predicted warming impacts for the uniform warming scenarios on maize yields are less negative when we use within-growing season weather variations. Our results emphasize the importance of careful modeling and estimation strategies in the context of assessing the climate change impacts.

We find that disaggregating the weather variables into monthly variables substantially increases the model performances. This is consistent with the most recent study that utilize the within-season variations^8^. Adding the interaction terms does not improve out-of-sample prediction performances when they are aggregated into the growing season total (comparing M2 with M1). Interaction terms only increase the model performances when we model weather variables with more finer timescales indicating that the within-growing season variation matters. Bayesian approaches in general provides more precise estimates.

We also observe that the conditional marginal effects of GDD and HDD differ across months within growing season. Combining such observation with the smaller predicted warming impacts from M4 compared to those from M1 and M2 suggests that the impacts of monthly temperature are not additive and growing-season aggregation may lead to overestimation of warming impacts.

Further researches on managing much larger numbers of weather variables to incorporate finer timescales within-season disaggregations or more flexible growing and heating degree days computations (e.g. different temperature thresholds across different time periods) would shed more light on the impacts of climate changes on crop production. Also, the current work can be extended to consider the role of improved technology^24^. Such researches would require developing more sophisticated statistical methods and estimation strategies.

## Methods

### Data and variables

Our dependent variable, corn yield, is obtained from the U.S. Department of Agriculture’s National Agricultural Statistics Service^25^. Corn yields data are available for almost all corn-producing counties, annually. We use the data from corn belt counties in Iowa, Illinois, and Indiana for the period of 1981–2017. The three states account for about 40% of the U.S. total maize production and face relatively similar production conditions. Total number of observations are 10,638.

We use the weather data from the Parameter-elevation Regressions on Independent Slopes Model (PRISM). PRISM is the widely-used interpolation procedure based on 2.5 × 2.5 mile grid system for the all contiguous states in the United States. Using the minimum and maximum temperatures from the PRISM data, we approximate a distribution of temperatures for each day based on a sinusoidal curve^1,21^ and then calculate the growing degree days (GDDs) and the heating degree days (HDDs) for each month, for the corn belt counties in Iowa, Illinois, and Indiana and for the period of 1981–2017. For the GDD calculation, we use the base temperature of 10 °C and the upper limit of 29 °C and for the HDD, we use the degree days above 29 °C. Monthly precipitation is simply the sum of daily precipitation from the PRISM dataset. Finally, we use state-specific quadratic time trends in all four models.

### Bayesian variable selection and model averaging

Let *γ* be one of the candidate models. Bayesian variable selection (BVS) can be done by finding the highest posterior probability of *γ*, that is, $\hat{\gamma }={\rm{\arg }}\,{{\rm{\max }}}_{\gamma }\,p(\gamma |{\rm{data}})$, where $p(\gamma |{\rm{data}})$ is the posterior probability that model *γ* is the true model given the data. From the Bayes’ theorem, the posterior model probability can be calculated by

$$p(\gamma |{\rm{data}})=\tfrac{p(\gamma )\,\int \,\int \,\int \,f(y|{\beta }_{\gamma },\alpha ,{\sigma }^{2})p({\beta }_{\gamma },\alpha ,{\sigma }^{2})d{\beta }_{\gamma }d\alpha d{\sigma }^{2}}{{\sum }_{\gamma }\,p(\gamma )\,\int \,\int \,\int \,f(y|{\beta }_{\gamma },\alpha ,{\sigma }^{2})p({\beta }_{\gamma },\alpha ,{\sigma }^{2})d{\beta }_{\gamma }d\alpha d{\sigma }^{2}},$$

where $p(\gamma )$ denotes the prior probability that *γ* is the true model, *β*_*γ*_ is the coefficient vector for the weather variables under model *γ*, *α* is the coefficient vector for the time trends and the fixed effects, and *σ*^2^ is the variance of the error term. Since we have no preferred model, it is reasonable to consider $p(\gamma )=1/{2}^{56}$, where 56 indicates the total number of weather-related variables. After some algebra, the posterior model probability reduces to

$$p(\gamma |{\rm{data}})=\frac{m({\rm{data}}|\gamma )}{{\sum }_{\gamma }\,m({\rm{data}}|\gamma )},$$

where

$$\begin{array}{rcl}m({\rm{data}}|\gamma ) & = & \Gamma (\frac{n-{p}_{\gamma }}{2})\,|{W}_{\gamma }^{\top }{W}_{\gamma }{|}^{-1/2}\\  &  & \times \,{[\pi {y}^{\top }\{{I}_{n}-{W}_{\gamma }{({W}_{\gamma }^{\top }{W}_{\gamma })}^{-1}{W}_{\gamma }^{\top }\}y]}^{-\frac{n-{p}_{\gamma }}{2}},\end{array}$$

*W*_*γ*_ = (*X*_*γ*_, *Z*), and *p*_*γ*_ is the number of columns in *W*_*γ*_. Hence, the best model $\hat{\gamma }$ is determined by maximizing *m*(data|*γ*).

To address the uncertainty associated with the estimated model $\hat{\gamma }$, Bayesian model averaging (BMA) uses

$$p(\theta |{\rm{data}})=\sum _{\gamma }\,p(\theta |{\rm{data}},\gamma )p(\gamma |{\rm{data}}).$$

We exclude the poor predictive models not belonging to

$${\mathscr A}=\{\gamma \in {\bf{\mathscr G}}:\frac{m(y|\hat{\gamma })}{m(y|\gamma )}\le c\},$$

where ${\bf{\mathscr G}}$ is a set of the simulated models from Markov chain Monte Carlo model composition (MC^3^)^26^ and *c* is some constant. We use *c* = 3. Hence, our BMA method is based on

$${p}_{{\mathscr A}}(\theta |{\rm{data}})=\sum _{\gamma \in {\mathscr A}}\,p(\theta |{\rm{data}},\gamma ){p}_{{\mathscr A}}(\gamma |{\rm{data}}),$$

where

$${p}_{{\mathscr A}}(\gamma |{\rm{data}})=\frac{m({\rm{data}}|\gamma )}{{\sum }_{\gamma \in {\mathscr A}}\,m({\rm{data}}|\gamma )}$$

is the modified posterior model probability. Further descriptions on Bayesian model specifications, posterior inferences, and optimization techniques can be found in the online Supplementary Information.

### Out-of-sample predictions

In order to test for the out-of-sample prediction performances, which is reported in Table 2, we conduct k-fold cross-validation with *k* = 10 for each specification. For this 10-fold cross-validation, we randomly split the sample into ten subsamples keeping the year blocks and end up having seven subsamples that consist of four years and three subsamples that consist of three years.

For specification *m*, our four measures of the out-of-sample prediction performances are defined as: $RMS{E}_{m}=\sqrt{MS{E}_{m}}$, $MAP{E}_{m}=\frac{100 \% }{N}\,\ast \,{\sum }_{n=1}^{N}\,|\frac{{y}_{n}-{y}_{nm}}{{y}_{n}}|$, $PC{C}_{m}=corr(y,{\hat{y}}_{m})$, and $Skill\,Score=-\,\frac{MS{E}_{m}-MS{E}_{M1}}{MS{E}_{M1}}$, where *N* is the number of observations, *y* is the actual values and ${\hat{y}}_{m}$ is the predicted values from specification *m* and *MSE*_*m*_ is the mean squared error of specification *m*. We report the average of these measures across all cross-validation iterations.

## Supplementary information

Supplementary Information

## References

[1] Schlenker W, Roberts MJ (2009). Nonlinear temperature effects indicate severe damages to us crop yields under climate change. Proceedings of the National Academy of Sciences.

[2] Urban D, Roberts MJ, Schlenker W, Lobell DB (2012). Projected temperature changes indicate significant increase in interannual variability of us maize yields. Climatic change.

[3] Butler EE, Huybers P (2013). Adaptation of us maize to temperature variations. Nature Climate Change.

[4] Schauberger B (2017). Consistent negative response of us crops to high temperatures in observations and crop models. Nature communications.

[5] Butler EE, Huybers P (2015). Variations in the sensitivity of us maize yield to extreme temperatures by region and growth phase. Environmental Research Letters.

[6] Tack, J., Barkley, A. & Nalley, L. L. Effect of warming temperatures on us wheat yields. *Proceedings of the National Academy of Sciences* 201415181 (2015). 10.1073/pnas.1415181112 PMC4460489 25964323

[7] Mourtzinis S, Ortiz BV, Damianidis D (2016). Climate change and enso effects on southeastern us climate patterns and maize yield. Scientific reports.

[8] Ortiz-Bobea A, Wang H, Carrillo CM, Ault TR (2019). Unpacking the climatic drivers of us agricultural yields. Environmental Research Letters.

[9] Lobell DB, Burke MB (2008). Why are agricultural impacts of climate change so uncertain? The importance of temperature relative to precipitation. Environmental Research Letters.

[10] Zipper SC, Qiu J, Kucharik CJ (2016). Drought effects on us maize and soybean production: spatiotemporal patterns and historical changes. Environmental Research Letters.

[11] Anderson CJ, Babcock BA, Peng Y, Gassman PW, Campbell TD (2015). Placing bounds on extreme temperature response of maize. Environmental Research Letters.

[12] Tack J, Barkley A, Hendricks N (2017). Irrigation offsets wheat yield reductions from warming temperatures. Environmental Research Letters.

[13] Carter EK, Melkonian J, Riha SJ, Shaw SB (2016). Separating heat stress from moisture stress: analyzing yield response to high temperature in irrigated maize. Environmental Research Letters.

[14] Tibshirani Robert (1996). Regression Shrinkage and Selection Via the Lasso. Journal of the Royal Statistical Society: Series B (Methodological).

[15] Wang, X., Dunson, D. & Leng, C. No penalty no tears: Least squares in high-dimensional linear models. In *Proceedings of the 33rd International Conference on International Conference on Machine Learning - Volume 48*, ICML’16, 1814–1822 (2016).

[16] Hoeting JA, Madigan D, Raftery AE, Volinsky CT (1999). Bayesian model averaging: a tutorial. Statistical Science.

[17] O’Hara RB (2009). A review of bayesian variable selection methods: what, how and which. Bayesian analysis.

[18] Narisetty NN, He X (2014). Bayesian variable selection with shrinking and diffusing priors. The Annals of Statistics.

[19] U.S. Department of Agriculture, National Agricultural Statistics Service. Field crops usual planting and harvesting dates. Retrieved from, http://usda.mannlib.cornell.edu/usda/current/planting/planting-10-29-2010.pdf (2010).

[20] Zhao C (2017). Temperature increase reduces global yields of major crops in four independent estimates. Proceedings of the National Academy of Sciences.

[21] Snyder RL (1985). Hand calculating degree days. Agricultural and forest meteorology.

[22] Cicchino M, Edreira J, Otegui M (2010). Heat stress during late vegetative growth of maize: effects on phenology and assessment of optimum temperature. Crop science.

[23] Wilhelm E, Mullen R, Keeling P, Singletary G (1999). Heat stress during grain filling in maize: effects on kernel growth and metabolism. Crop science.

[24] Huffman, W. E., Jin, Y. & Xu, Z. The economic impacts of technology and climate change: new evidence from us corn yields. *Agricultural Economics* (2018).

[25] U.S. Department of Agriculture, National Agricultural Statistics Service. NASS QuickStat. Retrieved from, https://quickstats.nass.usda.gov/ (2015).

[26] Madigan D, York J (1995). Bayesian graphical models for discrete data. International Statistical Review/Revue Internationale de Statistique.

