# Supplementary material for: Estimating non-additive within-season temperature effects on maize yields using Bayesian approaches
Source: Sci Rep. 2019 Dec 6;9:18566. doi: 10.1038/s41598-019-55037-6 (PMC6898414; doi:10.1038/s41598-019-55037-6)
Supplement: Supplementary file 1 — Supplementary Information [file 41598_2019_55037_MOESM1_ESM.pdf]

Supplementary Information for “Estimating non-additive within-season temperature effects on maize yields using Bayesian approaches"

Jisang Yu[1,*,+] **and Gyuhyeong Goh**[2,+]

[1]Department of Agricultural Economics, , Kansas State University , KS66506, USA
[2]Department of Statistics, , Kansas State University, , KS66506, USA
[*]Corresponding author: jisangyu@ksu.edu
[+]These authors contributed equally to this work

ABSTRACT

This supplementary material provides a) supplementary figures that describe weather data, b) technical details for the implementation of Bayesian Variable Selection (BVS) and Bayesian Model Averaging (BMA) for estimating Model 4 (M4) and c) out-of-sample prediction results of LASSO for M4.

1 Box plots of weather variables

Monthly weather variables have heterogenous distributions across months. For corn belt counties in Iowa, Illinois, and Indiana for the period of 1981 - 2017, Figure 1 represents the distributions of the growing degree days (GDDs) and the heating degree days (HDDs) for each month. As expected, HDD shows more heterogenous distributions across months compared to the distributions of GDD. Figure 2 represents the precipitation distribution for each month. With May and June being more wetter, other months show similar distributions. With such variations, if the temperature effects are not additive across different months, the predicted warming impacts from the growing season aggregated model would be different from the models that utilizing within-season variations.

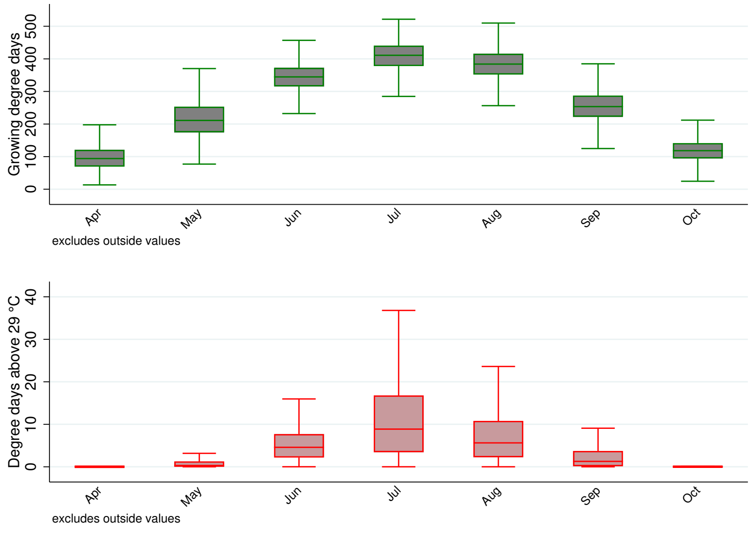

Figure 1. Degree days distribution by month

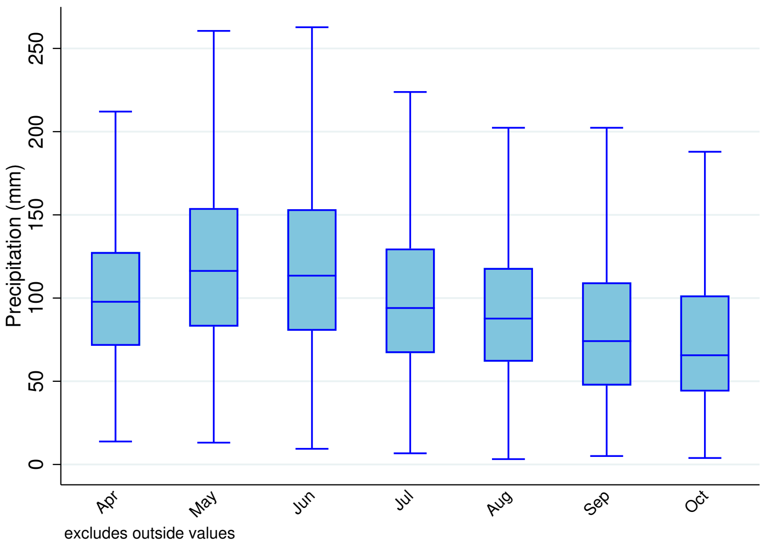

Figure 2. Precipitation distributions by month (mm)

2 Bayesian model specification and estimation

Let y_{it} and x_{it} be the logarithm of corn yield and a vector of the weather-related variables in county i and year t, respectively. Define the state indicator as D_s, i.e., $D_s = 1$ if the county belongs to state s and 0 otherwise. Similarly, we define the county indicator as C_i. Then, M4 can be expressed as

$$y_{it} = x_{it}^{\top}\beta + \sum_s \delta_{1s} D_s \times Time_t + \sum_s \delta_{2s} D_s \times Time_t^2 + \sum_i v_i C_i + \varepsilon_{it}, \tag{1}$$

where β is the coefficient vector for the weather variables and ε_{it} denotes the error term independently and normally distributed with mean 0 and variance σ^2. Let y be the response vector of log yields, X be the design matrix of weather variables, and Z be the design matrix of the state-specific quadratic time trends and county-level fixed effects. Then, Equation [1] can be re-written as

$$y = X\beta + Z\alpha + \varepsilon,$$

where α is the coefficient vector for the time trends and the fixed effects and ε follows the multivariate normal distribution with mean vector 0 and covariance matrix $\sigma^2 I$. Hence, the likelihood function is

$$f(y|\beta,\alpha,\sigma^2) = \frac{1}{(2\pi\sigma^2)^{n/2}} \exp\left(-\frac{1}{2\sigma^2}\|y - X\beta - Z\alpha\|^2\right),$$

where n denotes the length of the vector y. To complete our Bayesian model specification, we need to define the prior distribution about unknown parameters (α,β,σ^2). As we have no prior information available for α and σ^2, we consider a non-informative prior as follows:

$$p(\alpha,\sigma^2) \propto 1/\sigma^2.$$

In M4, our key assumption is that certain monthly weather variables are irrelevant to the log yields. To incorporate our assumption into the posterior inference, we employ the notion of the spike-and-slab prior[1]. Let β_j be the jth element of β. Define γ_j as the indicator of inclusion of the jth weather variable, that is, $\gamma_j = 1$ if the jth variable is active in the model and 0

otherwise. We now define $p(\beta) = \prod_j p(\beta_j)$ such that

$$p(\beta_j) \propto \gamma_j + (1-\gamma_j)\delta_0,$$

where δ_0 denotes a point mass distribution at 0. From the Bayes' theorem, the posterior distribution is proportional to

$$p(\beta, \alpha, \sigma^2 | \text{data}) \propto f(y|\beta, \alpha, \sigma^2) p(\alpha, \sigma^2) p(\beta).$$

Note that if $\gamma_i = 0$, then $\beta_j = 0$ with the posterior probability of 1 and thus the jth weather variable is excluded from our Bayesian model. Hence, for given γ, our Bayesian model reduces to

$$f(y|\beta_\gamma, \alpha, \sigma^2) = \frac{1}{(2\pi\sigma^2)^{n/2}} \exp\left(-\frac{1}{2\sigma^2}\|y - X_\gamma\beta_\gamma - Z\alpha\|^2\right),$$
$$p(\beta_\gamma, \alpha, \sigma^2) = 1/\sigma^2,$$

where β_γ and X_γ are a sub-vector of β and a sub-matrix of X corresponding to $\gamma_j = 1$, respectively.

2.1 Posterior inference

In our Bayesian framework, the posterior inference can be easily implemented by generating posterior samples. Using the Gibbs sampler, which is the most popular Markov Chain Monte Carlo (MCMC) technique, the posterior samples can be obtained by iterating sampling of the following full conditional distributions:

$$\beta_\gamma | \alpha, \sigma^2, \text{data} \sim \text{Normal}\left\{(X_\gamma^\top X_\gamma)^{-1} X_\gamma^\top (y - Z\alpha), \sigma^2 (X_\gamma^\top X_\gamma)^{-1}\right\}, \quad (2)$$

$$\alpha | \beta_\gamma, \sigma^2, \text{data} \sim \text{Normal}\left\{(Z^\top Z)^{-1} Z^\top (y - X_\gamma\beta_\gamma), \sigma^2 (Z^\top Z)^{-1}\right\}, \quad (3)$$

$$\sigma^2 | \alpha, \beta_\gamma, \text{data} \sim \text{Inverse}-\text{Gamma}\left(\frac{n}{2}, \frac{\|y - X\beta - Z\alpha\|^2}{2}\right), \quad (4)$$

where $\text{Normal}(\mu, \Sigma)$ denotes the multivariate normal distribution with mean vector μ and covariance matrix Σ, and $\text{Inverse}-\text{Gamma}(\text{a}, \text{b})$ denotes the inverse gamma distribution with shape parameter a and scale parameter b. The proof of Equations [2] – [4] can be easily shown by the Bayes' theorem.

2.2 Bayesian variable selection

Since γ represents one of the candidate models, Bayesian variable selection (BVS) can be done by finding the highest posterior probability of γ, that is, $\hat{\gamma} = \arg\max_\gamma p(\gamma|\text{data})$, where $p(\gamma|\text{data})$ is the posterior probability that model γ is the true model given data. From the Bayes' theorem, the posterior model probability can be calculated by

$$p(\gamma|\text{data}) = \frac{p(\gamma)\int\int\int f(y|\beta_\gamma, \alpha, \sigma^2) p(\beta_\gamma, \alpha, \sigma^2) d\beta_\gamma d\alpha d\sigma^2}{\sum_\gamma p(\gamma)\int\int\int f(y|\beta_\gamma, \alpha, \sigma^2) p(\beta_\gamma, \alpha, \sigma^2) d\beta_\gamma d\alpha d\sigma^2},$$

where $p(\gamma)$ denotes the prior probability that γ is the true model. Since we have no preferred model, it is reasonable to consider $p(\gamma) = 1/2^{56}$, where 56 indicates the total number of weather-related variables. After some algebra, the posterior model probability reduces to

$$p(\gamma|\text{data}) = \frac{m(\text{data}|\gamma)}{\sum_\gamma m(\text{data}|\gamma)},$$

where

$$m(\text{data}|\gamma) = \Gamma\left(\frac{n - p_\gamma}{2}\right) |W_\gamma^\top W_\gamma|^{-1/2} \left[\pi y^\top \{I_n - W_\gamma (W_\gamma^\top W_\gamma)^{-1} W_\gamma^\top\} y\right]^{-\frac{n-p_\gamma}{2}},$$

$W_\gamma = (X_\gamma, Z)$, and p_γ is the number of columns in W_γ. Hence, the best model $\hat{\gamma}$ is determined by maximizing $m(\text{data}|\gamma)$. However, this optimization is computationally too expensive, because we need to visit $2^{56} (\approx 7.2 \times 10^{16})$ values of γ. To address this issue, we use Markov chain Monte Carlo model composition (MC^3)[2]. The main idea of MC^3 is to construct a Markov chain with the invariant distribution $p(\gamma|\text{data})$. To construct the Markov chain, we define a neighborhood of γ, say $\text{nbd}(\gamma)$, which consists of the model γ itself and the set of models with either one variable more or less than γ. Then, MC^3 can be implemented as follows:

1. Set the initial model $\gamma^{(0)}$;
2. Repeat for $k = 0, 1, 2, \ldots$;
   (a) Generate γ^* by drawing a model from $\text{nbd}(\gamma^{(k)})$ with a simple random sampling.
   (b) Generate u from a uniform distribution on $[0, 1]$.
   (c) Update $\gamma^{(k+1)} = \gamma^*$ if
   $$u \le \min\left\{1, \frac{m(\text{data}|\gamma^*)}{m(\text{data}|\gamma^{(k)})}\right\}.$$
   Otherwise, stay $\gamma^{(k+1)} = \gamma^{(k)}$.

Using this MC^3 algorithm, we can easily determine the best model $\hat{\gamma}$ which is the most frequently generated model. Let $\gamma^{(1)}, \ldots, \gamma^{(M)}$ be the samples generated through MC^3. In our analysis, we generate $15{,}000$ samples after discarding $5{,}000$ burn-in samples. Define $\mathscr{G}$ as a set of the generated models. Then, we define the best model $\hat{\gamma}$ such that

$$\sum_{k=1}^{M} \text{I}(\hat{\gamma} = \gamma^{(k)}) \ge \sum_{k=1}^{M} \text{I}(\gamma = \gamma^{(k)})$$

for any $\gamma \in \mathscr{G}$, where $\text{I}(\cdot)$ is an indicator function.

2.3 Bayesian model averaging

It is worth noting that the posterior inference in BVS relies on $p(\theta|\text{data}, \hat{\gamma})$, where $\theta = (\beta, \alpha, \sigma^2)$ is the parameter and $\hat{\gamma}$ is the selected best model. To address the uncertainty associated with the estimated model $\hat{\gamma}$, Bayesian model averaging (BMA) uses

$$p(\theta|\text{data}) = \sum_{\gamma} p(\theta|\text{data}, \gamma) p(\gamma|\text{data}). \tag{5}$$

However, Madigan and Raftery[3] remark that if a model predicts the data far less well than the best model, then it should be excluded from Equation [5]. We also exclude the poor predictive models not belonging to

$$\mathscr{A} = \left\{\gamma \in \mathscr{G} : \frac{m(y|\hat{\gamma})}{m(y|\gamma)} \le 3\right\},$$

where $\mathscr{G}$ is a set of the simulated models from MC^3. Hence, our BMA method is based on

$$p_{\mathscr{A}}(\theta|\text{data}) = \sum_{\gamma \in \mathscr{A}} p(\theta|\text{data}, \gamma) p_{\mathscr{A}}(\gamma|\text{data}),$$

where

$$p_{\mathscr{A}}(\gamma|\text{data}) = \frac{m(\text{data}|\gamma)}{\sum_{\gamma \in \mathscr{A}} m(\text{data}|\gamma)}$$

is the modified posterior model probability.

2.4 Out-of-sample prediction of LASSO for M4

As we discuss in the main body, Penalized Least Squares approaches suffer from a challenge of choosing tuning parameters, which control the degree of the sparsity. Many studies show that Bayesian approaches are more effective in terms of out-of-sample predictions[4–6]. Here we report the out-of-sample performance of LASSO estimation for M4 and show it performs quite poorly.

Table 1. Out-of-sample prediction performances for LASSO for M4

Specifications	RMSE	MAPE	PCC	Skill Score
M4-LASSO	0.1577	0.0252	0.7889	-0.2159

References

1. Mitchell, T. J. & Beauchamp, J. J. Bayesian variable selection in linear regression. *J. Am. Stat. Assoc.* **83**, 1023–1032 (1988).
2. Madigan, D. & York, J. Bayesian graphical models for discrete data. *Int. Stat. Rev. Int. de Stat.* **63**, 215–232 (1995).
3. Madigan, D. & Raftery, A. E. Model selection and accounting for model uncertainty in graphical models using occam's window. *J. Am. Stat. Assoc.* **89**, 1535–1546 (1994).
4. Hoeting, J. A., Madigan, D., Raftery, A. E. & Volinsky, C. T. Bayesian model averaging: a tutorial. *Stat. Sci.* **14**, 382–417, DOI: 10.1214/ss/1009212519 (1999).
5. O'Hara, R. B., Sillanpää, M. J. *et al.* A review of bayesian variable selection methods: what, how and which. *Bayesian analysis* **4**, 85–117 (2009).
6. Narisetty, N. N. & He, X. Bayesian variable selection with shrinking and diffusing priors. *The Annals Stat.* **42**, 789–817, DOI: 10.1214/14-AOS1207 (2014).
